# Recovery of High Value-Added Compounds from Food By-Product

**DOI:** 10.3390/foods11121670

**Published:** 2022-06-07

**Authors:** Beatriz Gullón, Remedios Yáñez

**Affiliations:** 1 Department of Chemical Engineering, Faculty of Science, University of Vigo (Campus Ourense), As Lagoas, 32004 Ourense, Spain; 2 CINBIO—Centro de Investigacións Biomédicas, Universidade de Vigo, 36310 Vigo, Spain

The agri-food industry generates large quantities of by-products, both of animal and vegetable origin, which are currently discarded or destined to low-value-added applications [1]. However, they frequently present very interesting chemical compounds with great application potential in the pharmaceutical, food, or cosmetics industries. Therefore, to move towards a circular economy with a high level of resource efficiency, it would be necessary to develop integrated multi-product biorefineries based on environmentally friendly techniques that extract the most value from these by-products, a strategy that would minimize adverse effects on human health and the environment while improving the economic competitiveness of these sectors [2].

In this special issue, interesting valorization opportunities for several agri-food by-products are explored in 9 papers, by-products such as fruit wastes like melon peel [3], soybean [4] and chestnut [5] residues, alfafa [6], and fish [7] and meat remains [8]. Authors evaluate different green technologies for the recovery of several valuable fractions, such as proteins, carbohydrates, pectic oligosacharides, and phenolic compounds, and assess their potential as sustainable sources of bioactive compounds, analysing their antioxidant, antibacterial, anti-tyrosinase, and anti-inflammatory activities as well as their prebiotic potential. On the other hand, authors review the biological activities exerted by tannins extracted from by-products of the agri-food industry [9], the employment of innovative techniques involved in the conversion of rice bran into valuable food ingredients [10] and the application of agro-industrial wastes in aquaculture [11].
